# Regional technology gap and innovation efficiency trap in Chinese pharmaceutical manufacturing industry

**DOI:** 10.1371/journal.pone.0233093

**Published:** 2020-05-20

**Authors:** Hongbo Lai, Hao Shi, Yang Zhou

**Affiliations:** Business School, University of Shanghai for Science and Technology, Shanghai, China; Institute for Advanced Sustainability Studies, GERMANY

## Abstract

**Objective:**

There is a huge technology gap between regions in Chinese pharmaceutical manufacturing industry, which is the reality that must be faced. However, most of the available researches on innovation efficiency are based on the logic of a given technology level, ignoring the regional technological gap. This paper will stand from the perspective of technology gap and re-examine the innovation efficiency of pharmaceutical manufacturing industry in different regions of China and its impact on regional industrial competitiveness.

**Methods:**

We use the DEA-BCC input-oriented model to measure innovation efficiency of 28 provinces from the data of China's pharmaceutical manufacturing industry. The threshold model is constructed, with technology level as the threshold variable, innovation efficiency as the main explanatory variable, and industrial competitiveness as the dependent variable. In the threshold model, 28 regions are divided into three technical groups, and further, the impact of innovation efficiency on industrial competitiveness in different groups is analyzed and compared.

**Results:**

According to the empirical research results, an U-shaped efficiency trap has been found in Chinese pharmaceutical manufacturing industry, and the areas with medium technical level are at the bottom of the trap. The improvement of innovation efficiency does not necessarily promote the improvement of regional industrial competitiveness. Only in high-level and low-level technology groups, innovation efficiency has effectively promoted the improvement of industrial competitiveness. In addition, the intensity of R&D investment has a similar impact on industrial competitiveness.

**Conclusions:**

The findings suggest that, regions in the efficiency trap should strive to seek opportunities for industrial transformation and focus on the industrial transformation of new technology, new industry and new opportunities, instead of blindly pursuing R&D investment intensity and superstitious innovation efficiency. So as to free up innovation resources for high-quality technological innovation in other regions. In addition, the Chinese government should make use of its public hospital system to normalize and expand the centralized drug procurement and eliminate the low-quality innovation.

## Introduction

The SARS-CoV-2 has made the pharmaceutical manufacturing industry the focus of global attention. China's pharmaceutical industry has resumed production rapidly, ensuring the global supply of pharmaceutical products and playing an important role in the fight against the epidemic. But, that the export of Chinese pharmaceutical products is mainly based on APIs and intermediates. The state shows that China is still far away from the upstream of the global pharmaceutical manufacturing industry chain and the achievements of pharmaceutical research and innovation in the middle reaches of the industry chain are insufficient. The loss brought by the epidemic makes Chinese pharmaceutical manufacturing industry more deeply realize the necessity and urgency of independent innovation and high-quality innovation.

In recent years, with the development of economy, the demand for quality and quantity of medical consumption of Chinese people is increasing day by day. Meanwhile the R&D investment in China's pharmaceutical manufacturing industry is also increasing. According to the statistics of China's science and Technology Yearbook, from 2011 to 2018, China's total health expenditure increased from 2434.59 billion yuan to 5912.19 billion yuan, while the expenditure on research and test development of pharmaceutical manufacturing industry increased from 21.12 billion yuan to 58.09 billion yuan. However, the main R&D investment of China's pharmaceutical manufacturing industry remains in generics and APIs. And there is still a lack of achievements in the field of original research and innovative drugs. It can be seen that the industrial technology innovation requires not only the support of consumption demand and R&D funds, but also the innovation efficiency and quality.

The unbalanced development of China's regional pharmaceutical manufacturing industry determines that there are huge differences in innovation efficiency and quality among regions. In some advanced regions, the mode of technological innovation of pharmaceutical manufacturing industries, has gradually changed from introduction, digestion, absorption and re-innovation to integrated innovation and original innovation. But, in the backward areas of pharmaceutical industry, the means of technological acquisition are still mainly technology introduction and technological transformation. More importantly, medical resources and patients are still gathering in developed areas, which will further widen the technology gap. In other words, the technology gap will still exist for a long time, which is the reality that scholars and regional leaders must face. But unfortunately, the technology gap has not been taken seriously, in the available literature on innovation activities in pharmaceutical manufacturing industry. Therefore, it is of great theoretical and practical significance to measure and analyze the innovation efficiency of China's regional pharmaceutical manufacturing industry from the perspective of regional technology gap.

## Innovation efficiency and technology gap

### Innovation efficiency

Scholars achieve the goal of industrial innovation efficiency through the idea of Innovation workshop. Innovation efficiency is the effective utilization of innovation input factors. Previous works interprets technological innovation activities as a production process existing in the field of science and technology, aiming at creating new knowledge and applications [1]. Low input and high output in quantity is high efficiency, otherwise it is low efficiency. Scholars place industrial innovation activities in an imaginary innovation workshop. And the innovation workshop has become the research object [2]. By comparing the input and output of each innovation workshop, we can get the production efficiency of the workshop. Then the production efficiency of innovation workshop is regarded as the innovation efficiency of innovation subject.

To measure the efficiency as a relative value, we must build a comparison sequence. Arranging these workshops from different angle may lead to different results. Industrial and regional comparison are the major comparison sequences for measurement on innovation efficiency of industries. In the industrial comparison sequence, some scholars regard the whole industry as an innovation workshop, and use the number of industrial panels to calculate the industrial innovation efficiency directly. Some scholars set up special industrial comparison sequence according to the scale and property right structure of enterprises. In the regional comparison sequence, each region is regarded as an innovation workshop. By comparing the performance of these workshops, we can get a series of efficiency values. These relative values are placed on the right side of the equation as dependent variables to study the influence of other factors on innovation efficiency. For example, scholar study the specific impact of government support, geographical proximity, factor market distortion on industrial innovation efficiency [3].

In the studies of innovation efficiency, regional differences are mainly reflected in economic development and geographical position. Fen (2013) divided China into eight major economic zones and found that the areas with high green innovation efficiency are relatively developed coastal areas, while the areas with relatively backward economy in the northwest and the middle reaches of the Yellow River have low green innovation efficiency [4]. Liu (2016) studied the innovation efficiency of Chinese high-tech zones in 2012, and the research shows that the Yangtze River Delta and Pearl River Delta regions have the highest technological efficiency [5]. Qian (2019), explored the technology gap and its influencing factors in different nature of enterprises, and find that there are obvious technical gaps between state-owned and privately-owned groups [6].But the technical gaps mentioned in Dr. Qian's paper refers to the input-output conversion rate gap, which is still the efficiency gap. Scholars pay attention to the impact of various regional differences on the efficiency of industrial innovation, but ignore the technological characteristics and technological gap of the industry itself.

In the research of innovation efficiency, pharmaceutical manufacturing industry has not been specially treated. The pharmaceutical manufacturing industry in each province is regarded as an independent innovation workshop. Shao (2016) calculated and analyzed the innovation efficiency of Chinese pharmaceutical manufacturing industry in 2009–2013, and found that innovation efficiency is not positively related to regional economic strength [7]. Xiao (2018) found that in Chinese pharmaceutical manufacturing industry, the provinces with the highest average efficiency were Sichuan, Tianjin, Jilin, Hunan and Guizhou, while the provinces with the lowest average efficiency were Shanxi, Hebei, Shandong, Guangdong and Jiangxi [8]. When all regions are integrated, the whole picture of pharmaceutical industry will appear. Liu (2013) found that the innovation efficiency of Chinese pharmaceutical manufacturing industry is gradually improving, but it is still at a low level, and he suggested that t pharmaceutical enterprises should start with saving R&D capital investment [9].These studies also failed to pay attention to the differences in the technical level of pharmaceutical manufacturing in different provinces of China. And they all stopped at innovation efficiency, lacking further study on the impact of innovation efficiency on industrial development.

### Technology gap

Technology gap is considered to be the difference of technology level among economic entities and the reason why advanced economic entities gain monopoly profits. Advanced countries have a large reserve of knowledge, technology and industrial production capacity, which can invent new products before the underdeveloped countries. In the international trade research literature, technology gap is often linked with foreign direct investment, technology spillover, technology absorption, technology catch-up and other key words. Based on the technology gap theory, scholars, Ji (2011), Fu (2013), Sun (2017) fully discussed the impact of technology gap, technology progress path and other factors on the technology development of China [10–12].

The formation of technology gap is driven by technology accumulation. Xu (2012) defined technology accumulation as the progression of technical knowledge and achievements obtained in long-term production and innovation practice [13]. Song (2011) believed that technology accumulation level can be used to represent the technology level of enterprises and measure the technology gap between enterprises [14]. Zhang (2018) used the sum of patents obtained in the past to measure technology accumulation, which is characterized as the technology gap between enterprises [15]. It is an important means for enterprises to protect their own business interests to obtain intellectual property protection through patent application. The accumulation of the number of patents can represent the accumulation of the technological level of enterprises.

### A brief comment

Most of the researches on innovation efficiency ignore the technology gap between regions, but only consider the efficiency difference in quantity. A small number of studies highlight the imbalance of regional development by dividing economic regions. But, the way of dividing China into the east, the middle and the west does not really reflect the regional technology gap of pharmaceutical manufacturing industry.

Innovation workshop makes it possible to measure the efficiency of innovation activities. But the production efficiency of a single workshop can not replace the development of the whole factory. Technological innovation in pharmaceutical industry should cover the whole process from R&D to market. The ultimate goal of technological innovation is to promote industrial development. The research on industrial innovation activities should not stop at the innovation activities or confuse innovation efficiency with industrial development.

Therefore, this paper introduces the technology gap perspective in the traditional innovation efficiency measurement model, in order to explore the impact of innovation efficiency on industrial development.

### Current situation of regional technology gap

**Fig 1:**
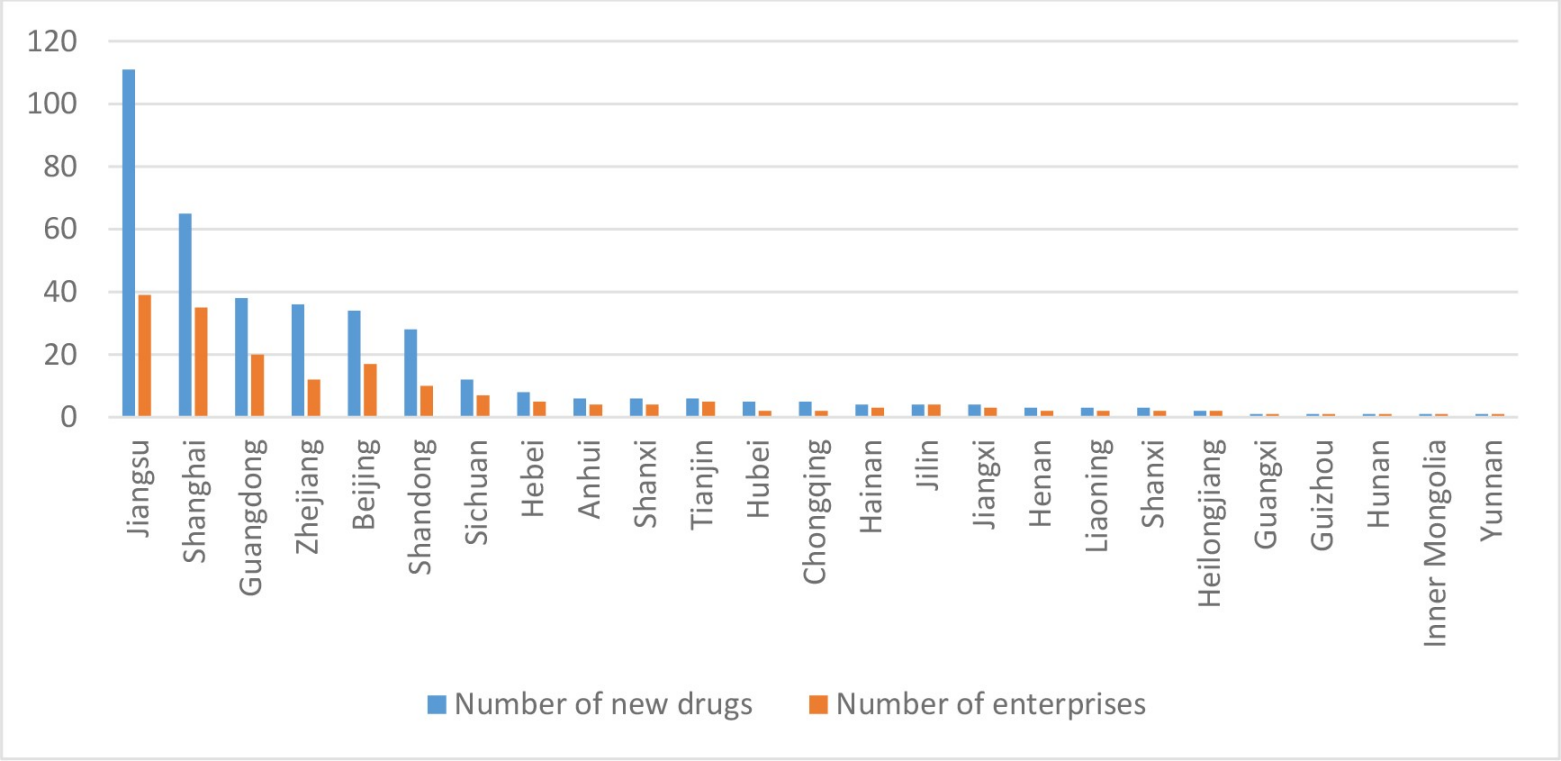
The situation of 1.1 or 1 class new chemicals approved for clinical use or on the market in various regions in 2007–2017.

In the pharmaceutical manufacturing industry of China, some developed regions have occupied the technological advantage, and have opened a distance with other regions. From Fig 1, we can see that in 2007–2017, after the concept of new drug management, the research and development achievements of new drugs meeting the new domestic standard and their affiliated enterprises are gathered in Jiangsu, Shanghai, Guangdong, Beijing, Zhejiang, Shandong and other regions. It can be seen that in terms of innovation ability and technology level, all regions have opened a considerable distance.

**Fig 2:**
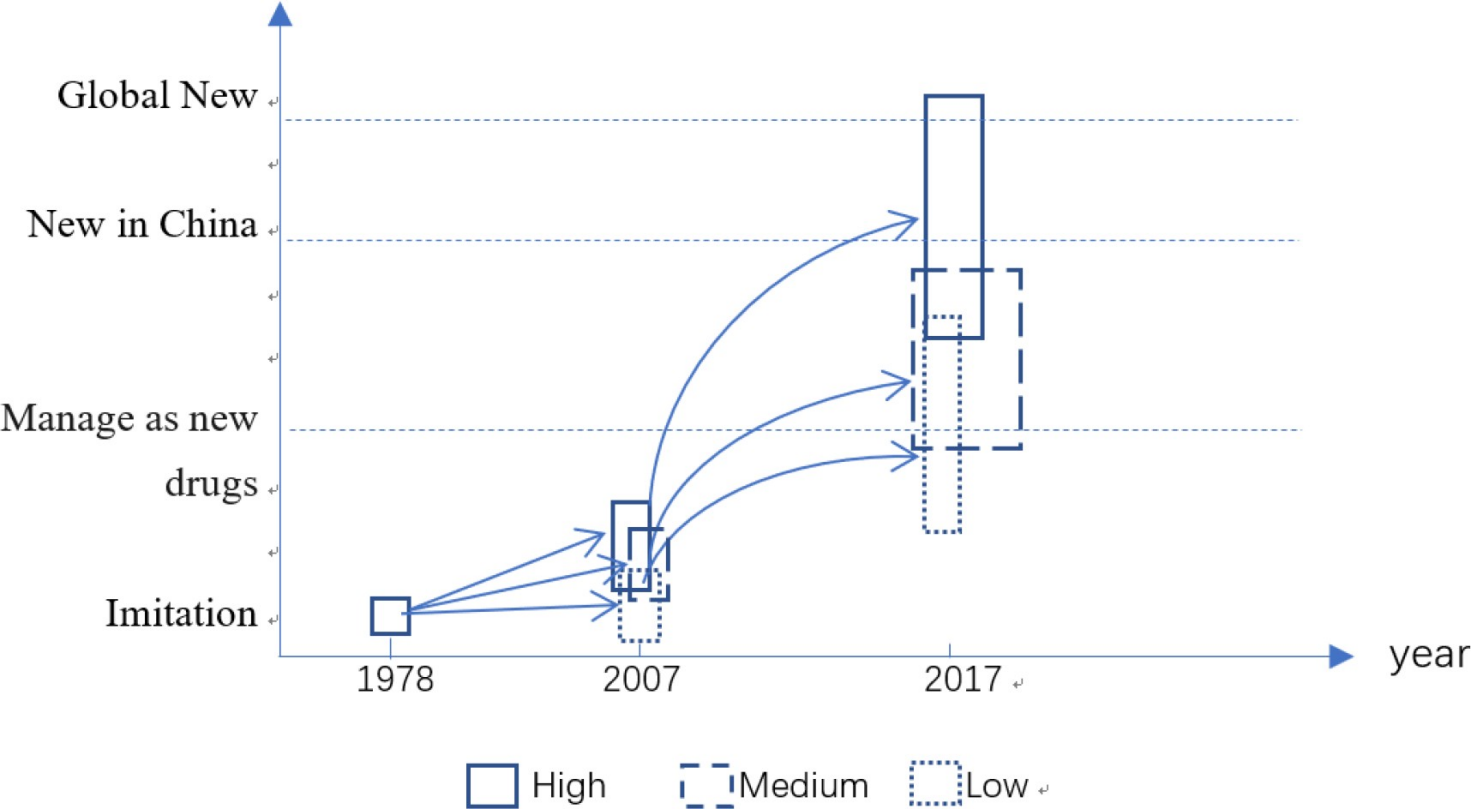
A sketch map of innovation level development in Chinese pharmaceutical industry from 1978 to 2017.

From Fig 2, we can more intuitively see the process of technology level pulling between regions. Since China implemented the reform and opening-up policy in1978, the national pharmaceutical industry started together, and all regions laid a quantitative foundation at the same level. Until 2007, the level of technology and economic development of pharmaceutical manufacturing industry in all regions had been improved, but the gap was not obvious. Subsequently, the impact of China's accession to the WTO on the pharmaceutical manufacturing industry is deepening day by day. Some regions stand out with the help of policy support, geographical location, traditional medicine and other comparative advantages. In 2017, some products of these high-tech areas have reached the world's leading level. No matter from the quantity or quality of research and development results, high-end regions have opened a gap with low-end regions. However, the gap between the middle and low-end regions is not obvious.

## Methodology and data

### DEA-BCC input-oriented model

DEA (data envelopment analysis) model is often used to measure the technical efficiency of production process [16]. For the following reasons DEA is suitable to analyze innovation performance: (1) DEA model is very effective in measuring the production efficiency of multi input and multi output production departments.(2) DEA does not assume the function form, and does not require judgment on the relative importance or weight of input and output.(3) DEA approach takes into consideration the complex nature of innovation and accommodates multiple inputs and outputs in a single analysis.

The choice of model orientation depends on the purpose of the analysis. If the purpose of the analysis is only to obtain the efficiency value of each unit, there is little difference between input-oriented and output-oriented. However, it is hoped that further discussion, such as the issue of efficient management, will be necessary to subdivide. If we take reducing input as the main way to adjust the efficiency of inefficiency unit, we should choose input orientation. If we take increasing output as the main way to improve efficiency, we should choose output-oriented model.

This research object of this paper is the innovation activities of the pharmaceutical manufacturing industry. Through the preliminary research on the innovation status and actual data of the pharmaceutical manufacturing industry in China, it is found that there is redundancy in the input of innovation elements in a large number of regions in China. Reducing the input will be the main way to improve efficiency, so the selection of input-oriented is better than output-oriented.

The basic assumption of BCC model is variable returns to scale [17]. Another common model is the CCR model, which assumes constant returns to scale. However, it is difficult for pharmaceutical manufacturing enterprises to maintain the optimal production scale for a long time. Therefore the assumption of BCC model is more realistic.

In this paper, the DEA-BCC input-oriented model is used to measure the production efficiency.

## Threshold model setting

Using threshold model, we can avoid the subjectivity of artificial grouping. According to Hansen (1999), the threshold of minimizing the sum of squares of residual errors is found by using the lattice search method [18]. Compared with the subjective differentiation method based on the geographical location or economic development level, this method can be more reasonable to distinguish the different regions according to the different technical level.

In this paper, the threshold regression model is constructed with the results of innovation efficiency measurement under the two common frontiers of incremental method and stock method as main explanatory variable, industrial competitiveness as the dependent variable, technological level as the threshold variable, and other influencing factors of industrial strength as independent variables.

The single threshold model is set as follows:

$${IC}_{it}=\mu_{i}+\theta x_{it}+\beta_{1}^{\prime }d_{it}I_{1}(q_{it}\leq \gamma )+\beta_{2}^{\prime }d_{it}I_{2}(q_{it}>\gamma )+\xi_{it}$$ (1)

The double threshold model is:

$${IST}_{it}=\mu_{i}+\theta x_{it}+\beta_{1}^{\prime }d_{it}I_{1}(q_{it}\leq \gamma_{1})+\beta_{2}^{\prime }d_{it}I_{2}(\gamma_{1}{<q}_{it}\leq \gamma_{2})+\beta_{3}^{\prime }d_{it}I_{3}(q_{it}>\gamma_{2})+\xi_{it}$$ (2)

*d_it_* is the main explanatory variable. *IC_it_* indicates the industrial competitiveness as dependent variable. *x_it_* is another independent variable, that is, the influencing factors of industrial development. *q_it_* is the threshold variable, which is set as technical level in this paper. I is a fictitious variable, if the qualifications are, I = 1, otherwise I = 0; I is the region, t is the year, $\theta ,\;\beta_{1}^{\prime },\;\beta_{2}^{\prime }$ is the coefficient to be estimated, *γ_i_* is the threshold value to be estimated, *μ_i_* is the individual intercept term, ξ_*it*_ is the random perturbation term. *i* indicates the area. *t* indicates the year.

## Database selection

Statistical yearbook is the official data of China, which has the advantages of integrity, availability and authority. Based on the statistical yearbook data, scholars have made a lot of reliable research on the development of various industries in China [19–20]. Another important database is the database of Chinese listed companies. This database mainly records the annual report data of listed companies. But, listed companies only account for a small part of Chinese pharmaceutical manufacturing enterprises. It is not suitable to use the data of listed companies to represent the development of regional industries. Therefore, we choose China Statistics Yearbook on High Technology Industry database as the data source of this paper.

We selected 28 provinces from the data of China's pharmaceutical manufacturing industry in 2009–2016. Due to insufficient data, Tibet, Xinjiang, Qinghai, Hong Kong, Macao and Taiwan are not included in the analysis. The data used in this paper can be obtain from *China Statistics Yearbook on High Technology Industry 2010–2017*. The annual statistical yearbook reflects the situation of the previous year. For example, the 2010 statistical yearbook reflects the situation in 2009.

### Variable selection

Innovation is regarded as the production activity of innovation workshop. In the innovation workshop, input factors can be simply divided into capital input and manpower input, while innovation output includes knowledge output and economic output.

(1) Capital input: new product R&D expenditure and internal R&D expenditure. For the sake of robustness, this paper uses incremental method and stock method, respectively, to analyze the two methods of capital accounting.

#### Incremental method

New product R&D expenditure [21]. Since the set increment was completely converted into output in that year, the price environment was consistent and no price reduction was made.

#### Stock method

R&D internal expenditure stock index [22]. The first step is to reduce the price of internal R&D expenditure based on 1998. The second step is to depreciate and accumulate the internal R&D expenditure over the years. The specific methods are as follows [23]:

$$K_{it}=(1‐\delta )K_{i,t‐1}+E_{it}\;(i=1,\;2\cdots \cdots n,\;t=1,\;2\cdots \cdots m)$$

Among them, i denotes region, t denotes time, K denotes capital stock, E denotes capital input, and depreciation rate δ takes 15%. Taking 1998 as the base period, this paper calculates the capital stock in 2009, other years and so on. Before calculating the capital stock, the R&D price index is used to reduce the internal expenditure of R&D funds and convert it into the fixed price in 1998. R&D Price Index = Fixed Asset Price Index *45%+Consumer Price Index *55% [24]. Price index data come from the Bureau of Statistics.

(2) Manpower input: full-time equivalent of R&D personnel. The full-time equivalence of R&D personnel is currently an internationally accepted indicator for comparative human resources investment in science and technology [25]. Some scholars regard the number of personnel in scientific and technological activities and R&D personnel as human input in scientific and technological research and development. However, this study considers that full-time equivalent of R&D personnel, through the method of converting workload, covers the contribution of R&D personnel and managers to innovation activities, and is closer to the actual situation of R&D activities in enterprises.

(3) Economic output: sales revenue of new products [26]. New product sales revenue is the direct result of enterprises participating in market competition through technological innovation. The output value of new products and sales revenue of new products are commonly used indicators to represent market returns, but after 2013, the output value of new products is not included in the statistical yearbook, so it is not selected. In the calculation of stock method, the price of new product sales revenue is adjusted based on 1998.

(4) Knowledge output: number of patent applications. The number of patents is the main indicator to characterize the technological level of industry at present, and the number of patent applications is the main form of realizing the statistical knowledge output of industrial innovation activities. Although from patent application to patent validity, there are still many uncertainties, and some enterprises are reluctant to apply for patents because of the confidentiality of knowledge [27]. Whether the number of patents can represent the output of knowledge is still controversial. However, due to the particularity of pharmaceutical manufacturing industry, patent application is still the most important means for pharmaceutical enterprises to seek protection of their rights and interests. The number of patent applications is an important indicator of pharmaceutical enterprises' innovation efforts.

(5) Technical Level (TE): Standardization of Number of valid invention patents. The regional comparison shows that the technical level of each region is a dynamic relative value. In this paper, the de-dimension normalization is carried out and the numerical values are standardized to the interval of [0.01,1]. The formulas are as follows: $\gamma_{ij}=\frac{a_{ij}-mina_{j}}{maxa_{j}-mina_{j}}*99+1$, a_ij_ is the number of valid invention patents in J years in I region, mina_j_ is the minimum value in J years series, maxa_j_ is the maximum value in J years, γ_ij_ is the standardized value.

(6) Industrial Competitiveness (IC): The regional pharmaceutical industry's industrial strength is characterized by the proportion of regional pharmaceutical owner's business income in the main business income of the whole industry in the country. IC = regional industry main business income / national total [28].

(7) Influencing factors of industrial development: R&D input (RD), characterized by total internal R&D expenditure, RD = log (internal R&D expenditure); R&D input intensity (RDIN), characterized by the proportion of internal R&D expenditure of regional industry in starting business income, RDIN = internal R&D expenditure/main business income; profit margin (PM) = profit/main business income; government support intensity (GSIN) = government funds in R&D expenditure/ total R&D funds [29]; extroversion dependence (ED) = new product exports / new product sales[30].

The descriptive statistics of the data involved in this paper are shown in Table 1.

**Table 1:** Descriptive statistical results of variables.

variable	Mean	sd	min	p25	p50	p75	max
IC	0.036	0.035	0.001	0.012	0.027	0.045	0.163
TE	0.289	0.269	0.01	0.099	0.198	0.366	1
RD	10.948	1.154	8.029	10.22	10.847	11.84	13.721
RDIN	0.017	0.009	0.002	0.009	0.016	0.023	0.044
PM	0.114	0.035	-0.029	0.09	0.111	0.132	0.223
GSIN	0.065	0.038	0.006	0.043	0.058	0.081	0.389
ED	0.073	0.089	0	0.013	0.047	0.094	0.507

## Empirical results and discussion

### Analysis on innovation efficiency

Based on the DEA-BCC input-oriented model, the efficiency of innovation activities in the pharmaceutical industry in 2009–2016 was measured and decomposed. The results and descriptive statistics of efficiency measurement using incremental capital input method are listed in Table 2.

**Table 2:** Descriptive statistics of innovation efficiency.

region	Crste				Region	Crste			
	mean	Sd	min	max		mean	sd	min	max
Anhui	0.74	0.14	0.50	1.00	Jiangsu	0.65	0.07	0.53	0.72
Beijing	0.60	0.12	0.43	0.79	Jiangxi	0.77	0.23	0.40	1.00
Fujian	0.55	0.11	0.34	0.68	Liaoning	0.49	0.14	0.24	0.64
Guangdong	0.51	0.11	0.36	0.68	Inner Mongolia	0.57	0.29	0.25	0.97
Gansu	0.35	0.19	0.12	0.61	Ningxia	0.90	0.12	0.72	1.00
Guangxi	0.69	0.27	0.30	1.00	Shandong	0.74	0.17	0.49	1.00
Guizhou	0.85	0.15	0.61	1.00	Shanxi	0.57	0.22	0.32	0.96
Hainan	0.65	0.17	0.39	0.92	Shaanxi	0.52	0.10	0.34	0.65
Hebei	0.50	0.14	0.27	0.66	Shanghai	0.65	0.19	0.37	1.00
Henan	0.55	0.09	0.47	0.71	Sichuan	0.84	0.22	0.51	1.00
Heilongjiang	0.34	0.16	0.18	0.62	Tianjin	0.99	0.03	0.91	1.00
Hubei	0.80	0.14	0.63	1.00	Yunnan	0.60	0.11	0.48	0.80
Hunan	0.87	0.22	0.49	1.00	Zhejiang	0.70	0.17	0.41	0.83
Jilin	0.69	0.12	0.50	0.85	Chongqing	0.87	0.15	0.63	1.00

From the column of maximum value, there are 12 areas that reach the frontier of production efficiency (crste) in the 8-year observation period (when the observation value is 1, it is considered to reach the frontier). We can find that although there is a gap in the development level of pharmaceutical industry in different regions, the efficiency of innovation activities in backward regions can also reach the forefront of efficiency through the effective allocation of innovation resources.

**Fig 3:**
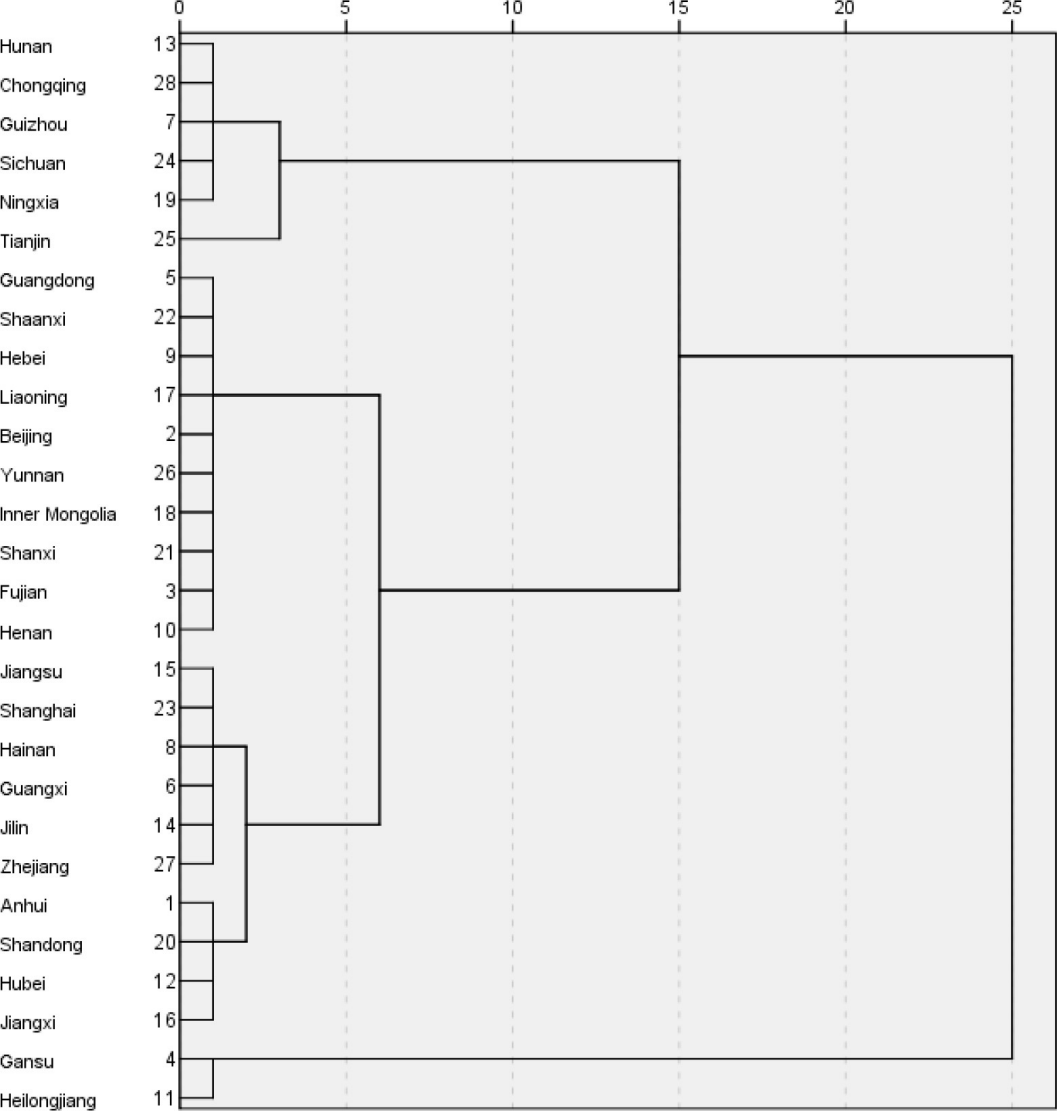
Cluster pedigree map of innovation efficiency.

Using the method of system clustering analysis, 28 decision units are classified according to the mean value of production efficiency (crste) from 2009 to 2016. The results are shown in Fig 3. Cluster analysis results are more detailed, but because classification is not the focus of this paper, the decision-making units are divided into three categories: high, medium and low according to the clustering pedigree map. The specific results are listed in Table 3.

**Table 3:** Classification results of innovation efficiency.

Category	Region
High efficiency	Hunan, Chongqing, Guizhou, Sichuan, Ningxia, Tianjin
Medium efficiency	Guangdong, Shaanxi, Hebei, Liaoning, Beijing, Yunnan, Inner Mongolia, Shanxi, Fujian, Henan, Jiangsu, Shanghai, Hainan, Guangxi, Jilin, Zhejiang, Anhui, Shandong, Hubei, Jiangxi
Poor efficiency	Gansu, Heilongjiang

From Table 3, it can be clearly seen that most of the regions are concentrated in the middle-efficiency group. In the high efficiency group, there are only six provinces, namely Hunan, Chongqing, Guizhou, Sichuan, Ningxia and Tianjin. In the low efficiency group, there are only two provinces, Gansu and Heilongjiang. The decision-making units of the high-efficiency group are geographically dispersed. While the low-efficiency group is only in the western and northeastern provinces. The result of clustering did not show the geographical distribution pattern of middle, east and west. Moreover, efficiency grouping does not fully reflect the technical level of each region. Guizhou and Ningxia are representatives of low technology level but high efficiency. It can be seen that low input and low output may also have high efficiency.

### The impact of innovation efficiency

This paper constructs a threshold model with industrial competitiveness as the dependent variable, technological level as the threshold variable, and innovation efficiency as the independent variable. Because of the existence of redundant parameters in the model, F statistics do not conform to the standard distribution, so Hansen proposed to calculate the critical value of F statistics by Bootstrap. This paper examines the significance of the single and double thresholds of innovation efficiency under two kinds of capital input statistics. The self-sampling P values of incremental method and stock method are shown in Tables 4 and 5 respectively.

**Table 4:** Threshold effect test of incremental method.

Critical value						
Model	F	P	BS	1%	5%	10%
Single threshold	13.872***	0.003	300	10.771	7.424	5.687
Double Threshold	9.365***	0.000	300	5.392	0.775	-0.601

Both P value and critical value are obtained by repeated sampling of 300 times by self-sampling method

***,** and * are significant at the levels of 1%, 5% and 10%, respectively, same as other tables.

**Table 5:** Threshold effect test of stock method.

Critical value						
Model	F	P	BS	1%	5%	10%
Single threshold	20.327***	0.003	300	15.170	11.425	8.356
Double Threshold	8.251***	0.000	300	3.002	0.343	-0.846

**Fig 4:**
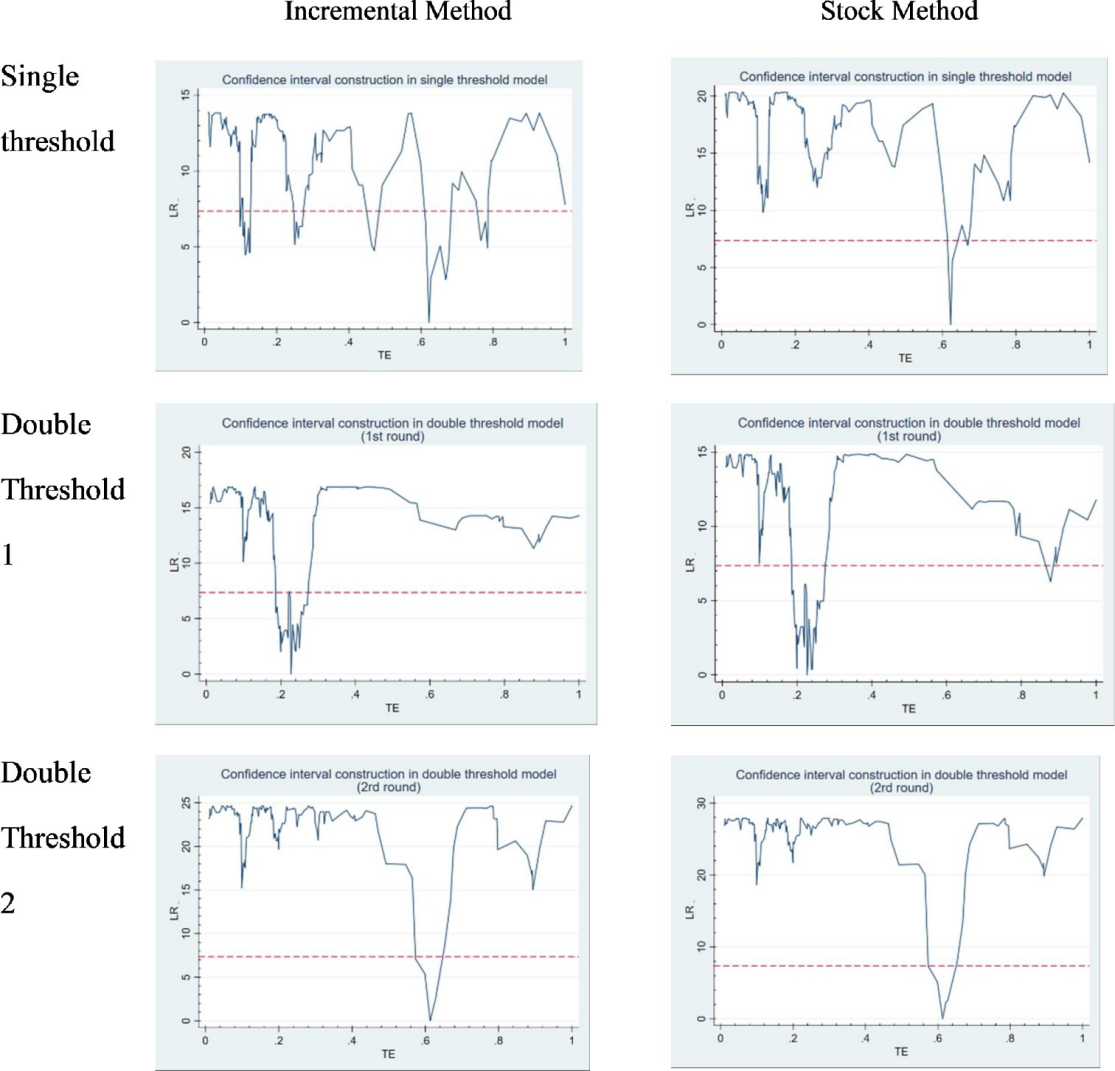
Likelihood ratio function graph for threshold effect test.

In Fig 4, two sets of likelihood ratio function graphs are presented to illustrate the process of estimating threshold values and constructing confidence intervals in a single threshold model. As shown in the figures, when the likelihood ratio test statistic (LR) is zero, the value of the variable TE is the estimated value of the threshold parameter. The critical value is 7.35 when the dotted line is less than 5% significant level, and the intersection of dotted line and LR curve is 95% confidence interval of threshold estimation. Tables 6 and 7 show the threshold values and 95% confidence intervals of the two sets of equations respectively.

**Table 6:** Threshold estimation results and confidence intervals of incremental method.

	Threshold Estimate	95% confidence interval
Single Threshold Model:	0.622	[0.098, 0.786]
Double Threshold Model:		
Ito1(g1)	0.227	[0.187, 0.271]
Ito2(g2)	0.613	[0.573, 0.627]

**Table 7:** Threshold estimation results and confidence intervals of stock method.

	Threshold Estimate	95% confidence interval
Single Threshold Model:	0.622	[0.622, 0.669]
Double Threshold Model:		
Ito1(g1)	0.227	[0.187, 0.878]
Ito2(g2)	0.613	[0.573, 0.627]

By comparing the two groups in Table 6 and Table 7, we can see some significant and robust results. When choosing a single threshold, the incremental method and the stock method respectively T = 0.622 and T = 0.227; when choosing double threshold, the incremental method successively determines the first course TE = 0.227 and the second threshold TE = 0.613. Although the order is different, the stock method also chooses TE = 0.227 and TE = 0.613 as two thresholds. The results show that the two thresholds have strong robustness. It is reliable and reasonable to divide 28 observations into three groups with 0.227 and 0.622.

Table 8 shows the frequency of occurrence of each region in the three groupings, bracketed for 2009–2016, and within eight years, the frequency of occurrence of the region in the three groupings. According to the two threshold values of the double threshold module, 28 regions were divided into three groups according to the technical level, low-end group (TE < 0.227), middle-end group (0.227 < TE < 0.613) and high-end group (TE > 0.613). From the perspective of grouping, Chinese pharmaceutical manufacturing industry is generally pyramid structure. In the high-end group, Shandong, Jiangsu, Guangdong, Tianjin and Zhejiang are the major traditional pharmaceutical provinces in China; in the middle-end group, Beijing, Shanghai, Sichuan and other areas are close to the upper limit of grouping; in the low-end group, the decision-making units are the most.

**Table 8:** Frequency statistics table of regional grouping by technology gap.

	Region
High	Shandong (8), Jiangsu (8), Guangdong (8), Tianjin (7), Zhejiang (5)
Middle	Tianjin (1), Zhejiang (3), Beijing (7), Shanghai (8), Hebei (8), Sichuan (6), Anhui (5), Hunan (5), Hubei (4), Yunnan (4), Guangxi (2), Jiangxi (2), Guizhou (2), Chongqing (2), Jilin (1), Liaoning (1)
Low	Beijing (1), Sichuan (2), Anhui (3), Hubei (3), Yunnan (4), Hunan (5), Guangxi (6), Jiangxi (6), Guizhou (6), Chongqing (6), Liaoning (7), Jilin (7), Hainan (7), Henan (8), Heilongjiang (8), Shanxi (8), Shaanxi (8), Fujian (8), Ningxia (8), Gansu (8), Inner Mongolia (8)

The numbers in brackets show the frequency of the region within the group in 8 years.

The regression results are listed in Table 9. The first is the regression results of fixed effect of non-threshold panel; the second is the regression results of incremental threshold model, with innovation efficiency as the main explanatory variable; the third is the regression model of fixed effect of non-linear panel based on the existing threshold, with R&D intensity as the main explanatory variable; and the fourth is the regression results of threshold model of stock method, with innovation efficiency as the main explanatory variable.

**Table 9:** The impact of innovation efficiency on the industrial competitiveness.

IC	Incremental method			Stock method Threshold Model
	Fixed-effect model	Threshold Model 1	Threshold Model 2	
TE	0.0084** (2.07)	0.0091**(2.03)	0.0084*(1.95)	0.0076(1.64)
RD	0.0047 *** (5.59)	0.0046***(5.59)	0.0047***(5.75)	0.0041***(5.16)
crste	-0.0043**(-2.07)		-0.0043**(-2.14)	
RDIN	-0.6828***(-6.08)	-0.6733***(-6.20)		-0.6186***(-5.80)
PM	-0.0452***(-3.20)	-0.0458***(-3.46)	-0.0436***(-3.27)	-0.0453***(-3.42)
GSIN	0.0135(1.22)	0.0133(1.24)	0.0116(1.09)	0.0127(1.18)
ED	0.0143**(2.25)	0.0111*(1.79)	0.0106*(1.71)	0.0116*(1.87)
crste_1		0.0027*(1.81)		0.0022(1.56)
crste_2		-0.0067***(-2.96)		-0.0045**(-2.07)
crste_3		0.0101***(3.30)		0.0123***(3.82)
RDIN_1			0.1489**(2.05)	
RDIN_2			-0.8183***(-7.00)	
RDIN_3			0.3140***(3.41)	
R^2^	0.6206	0.7081	0.7081	0.7157
F	7.58***	7.98***	8.15***	7.82***

The numbers in brackets are t statistics.

The regression results of influencing factors are explained as follows:

(1) There is a U-shaped efficiency trap. The improvement of innovation efficiency can promote the industrial competitiveness of the high-end group and the low-end group, while the situation of the middle-end group is just the opposite. From the regression results, that there is no significant difference between columns 2 and 4. Both the high-end group and the low-end group show a significant positive correlation between innovation efficiency and industrial competitiveness, while the middle group shows a significant negative correlation.

From the column 3, we can see that the intensity of R&D investment is similar. In threshold model 2, the regression coefficient of R&D investment intensity is significantly positive in high-tech areas and low-tech areas, but significantly negative in the middle end areas. In other words, the improvement of R&D investment intensity can not promote the enhancement of industrial strength in all regions. In the middle-end group, it only consumes limited R&D resources and does not effectively enhance the industrial competitiveness without considering the increase of business income.

It can be seen that middle-end group is at the bottom of a U-shaped efficiency trap, in which the promotion of innovation efficiency or R&D investment intensity can not enhance the industrial competitiveness.

(2) In all models, technology level and R&D investment are positively correlated with industrial competitiveness. It shows that technology level is the dominant force in the development of pharmaceutical manufacturing industry, and reasonable R&D investment can continuously promote industrial development.

(3) Profit margin has a significant negative correlation with industrial strength. Because of the huge investment in short-term scientific research in high-tech industry, it will appear in the form of cost in assets and liabilities, thus occupying profits. It can be seen that the profit growth of high-tech industry is not synchronized with industrial development.

(4) The influence of government support intensity on industrial development is not significant. It can also be found from the statistics that in R&D internal expenditure, government funds only account for a very small proportion compared with enterprise capital investment.

(5) The impact of industrial extroversion on industrial development is significantly positive. This shows that expanding sales and strengthening technical exchanges through opening up foreign markets can effectively improve industrial competitiveness.

## Discussion

(1) We affirm the importance of paying attention to regional technological differences when studying industrial innovation activities. The technology gap exists not only among the countries, but also in various regions of a country. There are not only innovation efficiency differences between regions, but also technology gap. The perspective of technology gap will bring more realistic results for the measurement of innovation efficiency and the research of innovation activities.

Technological level and innovation efficiency synergistically influence the development of pharmaceutical manufacturing industry. Technological level is the sustainable driving force for industrial development and it is also the key threshold for regional industry to leap from the low- or middle-end to the high-end. The relationship between innovation efficiency and industrial development shows a U-shape. Innovation efficiency is positively related to industrial development when the innovation efficiency is on the high or low level, but is negatively relate to industrial development when it is on the middle level, which we called the efficiency trap. Excessive pursuit of quantitative innovation efficiency and unlimited increase in R&D investment is not always a panacea. One prescription would never solve all the problems.

Advanced regions have already crossed the high threshold of technology accumulation. They have occupied the siphon effect on abstracting talents and investments. Meanwhile they also occupied the spillover effect on external technology. The industrial technology has formed a good self-reinforcing closed-loop effect in the continuous cycle of learning, using and innovating. On this basis, the efficiency of innovation activities and R&D investment increased. The increase in intensity will naturally promote industrial growth and continue to expand its advantages. Shandong, Jiangsu, Guangdong, Tianjin, Zhejiang and other regions, as large traditional pharmaceutical provinces, have a strong attraction for domestic high-tech talents, venture capital, and foreign advanced technology, but also have a better learning and absorption capacity than other regions.

The entrant regions have late entry advantage, so they have the choice to undertake the industrial transfer in the advanced regions according to their own resource endowment characteristics. On this basis, the efficiency of innovation activities and the increase of R&D investment intensity also properly promote the development of high-tech industries in the entrant regions from scratch.

(2) The paper points out a U-shaped efficiency trap of pharmaceutical manufacturing industry for policy makers. In addition, we provide a new model for decision makers to observe whether there are the same efficiency traps in other industries.

Medium-tech areas are in the efficiency trap. As the empirical results show, the efficiency of innovation activities and the intensity of R&D investment in medium-sized areas have not significantly promoted the increase of industrial sales share. Although the industrial development in all regions is difficult, compared with the other two types of regions, the middle-developed regions need to face more dilemmas. As the industrial technology level in the middle-developing areas has been separated from the low-end and has a large scale of production, its requirements for technological innovation are consistent with those in the high-tech areas. However, in the actual technological competition, high-tech areas occupy the advantages of manpower, capital and technology. It is difficult for middle-developed areas to achieve large-scale breakthroughs in core technology, and their R&D output can hardly form a real core competitiveness.

### Limitations of this study

(1) Market share is an important manifestation of industrial competitiveness, but it still can't show the whole picture of regional pharmaceutical manufacturing competitiveness. In the follow-up research, we will focus on refining the competitiveness indicators.

(2) Due to the limitation of data sources, the knowledge output of innovation activities is expressed in the form of the number of patents. Although the rationality is enhanced by grouping the technical level, the accumulation of the number is still difficult to fully reflect the market value of patents, and more difficult to reflect the quality gap between patents.

### Policy suggestion

Chinese government should eliminate low-quality innovation in medium-sized technology areas, by giving full play to the premium advantage of the public hospital system. In China's medical system, public hospitals occupy an absolute share and have a full bargaining advantage. Through the alliance of big cities or provinces, a bargaining alliance is formed, in which the low price of pharmaceutical manufacturers is exchanged for the purchase volume, and the high price enterprises are eliminated by means of competitive bidding. At the same time, take the consistency evaluation of generic drugs to ensure the quality of generic drugs. At this time, there are three ways out for pharmaceutical enterprises. First, carry out high-quality innovation and focus on the opening up of original research drugs. Second, expand production on a large scale to create low prices. Third, give up competition and exit the pharmaceutical industry. The rise of indigenous innovative drugs. Under the pressure of such policies, the cliff of pharmaceutical patents, which is prevalent all over the world, will soon appear in China.

Regions in the efficiency trap should strive to seek opportunities for industrial transformation. The transformation of pharmaceutical manufacturing industry will bring pain to local economic development. However, in industries without comparative advantages, blindly pursuing R&D investment intensity and superstitious innovation efficiency only produces tasteless R&D output, does not substantially improve industrial strength, and wastes precious innovation resources and fleeting development opportunities. Guizhou big data industry is a successful case of combining regional resource endowment with new industry and new technology. Therefore, it is imperative for the region with medium technology level to abandon the traditional innovation and development thinking of emphasizing quantity and light quality, avoid the efficiency trap in technological innovation, and focus on the industrial transformation of new technology, new industry and new opportunities.

## Supporting information

S1 Sheet Data set. (XLSX)

S1 Zip Analysis process. (ZIP)

S1 Text Database description. (DOCX)

S2 Text Explanation of input-oriented BCC model. (DOCX)
