# Supplementary material for: Regional technology gap and innovation efficiency trap in Chinese pharmaceutical manufacturing industry
Source: PLoS One. 2020 May 20;15(5):e0233093. doi: 10.1371/journal.pone.0233093 (PMC7239465; doi:10.1371/journal.pone.0233093)
Supplement: S1 Text — (DOCX) [file pone.0233093.s003.docx]

S3 Text. Database description

In this study, the pharmaceutical manufacturing industry in various regions of China is taken as the research object. From the perspective of authority, long-term, integrity, systematic, pertinence and pertinence, China Statistics Yearbook on High Technology Industry is selected as the data base.

**Authority:**

China Statistics Yearbook on High Technology Industry is compiled by the Statistics Bureau of the people's Republic of China. The National Bureau of statistics of the people's Republic of China, as an institution directly under the State Council, is in charge of national statistics and national economic accounting, formulates laws and regulations on statistical work, plans for statistical reform and modernization, and plans for national statistical investigation, organizes, leads, supervises and inspects the work of statistics and national economic accounting in various regions and departments, and supervises and inspects the implementation of statistical laws and regulations.

**Integrity:**

China Statistics Yearbook on High Technology Industry collects data on production and operation, R&D and related activities, and fixed asset investment of China's high technology industry in 2000-2016.

**Systematic:**

China Statistics Yearbook on High Technology Industry describes the basic situation of China's high-tech industry development in a more comprehensive way, and is the main reference book for relevant management departments and all sectors of the society to understand the development of China's high-tech industry. The content is divided into five parts. The first part mainly reflects the production and operation of high-tech industrial enterprises. The second part mainly reflects the R&D activities, new product development and sales, patents, technology acquisition and transformation, enterprises run R&D institutions, etc. The third part mainly reflects the fixed assets investment of high-tech industry enterprises. The fourth part is the international comparison data, which is sorted out according to the statistics of high-tech industry published by the world bank and other international organizations. The fifth part is appendix, including high-tech industry (manufacturing industry) classification (2013), high-tech industry (manufacturing industry) statistical classification catalog, high-tech industry (manufacturing industry) statistical data sorting and publishing format and main statistical index interpretation.

**Pertinence:**

1. China Statistics Yearbook on High Technology Industry mainly includes six general industries: pharmaceutical manufacturing, aerospace and equipment manufacturing, electronic and communication equipment manufacturing, computer and office equipment manufacturing, medical equipment and instrument manufacturing, and information chemicals manufacturing. Among them, the pharmaceutical manufacturing industry is divided into chemical manufacturing, proprietary Chinese medicine manufacturing and biological medicine manufacturing.

2. According to China Statistics Yearbook on High Technology Industry, the eastern region is divided into Beijing, Tianjin, Hebei, Shanghai, Jiangsu, Zhejiang, Fujian, Shandong, Guangdong and Hainan; the central region includes Shanxi, Anhui, Jiangxi, Henan, Hubei and Hunan; the western region includes inner Mongolia, Guangxi, Chongqing, Sichuan, Guizhou, Yunnan, Tibet, Shaanxi, Gansu, Qinghai Ningxia and Xinjiang; Northeast China includes Liaoning, Jilin and Heilongjiang. Hong Kong, Macao and Taiwan are not included in the statistical scope.

**Availability:**

China Statistics Yearbook on High Technology Industry is published by China Statistics Press.
