# Supplementary material for: Regional technology gap and innovation efficiency trap in Chinese pharmaceutical manufacturing industry
Source: PLoS One. 2020 May 20;15(5):e0233093. doi: 10.1371/journal.pone.0233093 (PMC7239465; doi:10.1371/journal.pone.0233093)
Supplement: S2 Text — (DOCX) [file pone.0233093.s004.docx]

**The Explanation of Choosing the Input Oriented BCC Model**

The choice of model orientation depends on the purpose of the analysis. If the purpose of the analysis is only to obtain the efficiency value of each unit, there is little difference between the two methods. However, it is hoped that further discussion, such as the issue of efficient management, will be necessary to subdivide.

If we take reducing input as the main way to adjust the efficiency of inefficiency unit, we should choose input orientation. If we take increasing output as the main way to improve efficiency, we should choose output-oriented model.

The research object of this paper is the innovation activities of the pharmaceutical manufacturing industry. Through the preliminary research on the innovation status and actual data of the pharmaceutical manufacturing industry in China, it is found that there is redundancy in the input of innovation elements in a large number of regions in China. Reducing the input will be the main way to improve efficiency, so the selection of input-oriented is better than output-oriented.

Compared with the assumption that the scale return of CCR model is constant, many production units are not in the optimal scale production state, so the assumption that the scale return of BCC model is variable is more realistic.

To sum up, this study chooses the input-oriented BCC model.

The specific algorithm of the model is as follows:

1、The basic principle of DEA method

With n Decision-Making Units $DMU_{j}(j=1,2,\cdots,n)$，Their input and output vectors are：$X_{j}=(x_{1j},x_{2j},\cdots,x_{mj})^{T}>0,$，$Y_{j}=(y_{1j},y_{2j},\cdots,y_{sj})^{T}>0, j=1,\cdots,n$.

Suppose the weight vectors of input and output are respectively $v=(v_{1},v_{2},\cdots,v_{m})^{T}$and$u=(u_{1},u_{2},\cdots,u_{s})^{T}$.

$\theta_{j}=\frac{u^{T}Y_{j}}{v^{T}X_{j}}=\frac{\sum_{r=1}^{s} u_{r}y_{rj}}{\sum_{i=1}^{m} v_{i}x_{ij}}, (j=1,2,\cdots n)$ is the efficiency evaluation index of $DMU_{j}(j=1,2,\cdots,n)$.

By changing $u$and $v$ as much as possible, make the maximum$\theta_{o}$. In order to measure the values of $\theta_{o}$, Charnes et al. Proposed the following CCR model in 1978:

The basic formula of input oriented CCR model：

$$Maximize\quad\frac{\sum_{r=1}^{s} u_{r}y_{ro}}{\sum_{i=1}^{m} v_{i}x_{io}}=\theta_{o}$$

$$subject to\quad\frac{\sum_{r=1}^{s} u_{r}y_{rj}}{\sum_{i=1}^{m} v_{i}x_{ij}}\leq1,j=1,2,\cdots,n,$$

$$\quad\quad\quad\quad\quad u_{r}\geq0,v_{i}\geq0, \forall r,i.$$

Input oriented CCR model：

$$Maximize \sum_{i=1}^{m} u_{r}y_{rj}$$

$$subject to \sum_{r=1}^{s} u_{r}y_{rj}-\sum_{i=1}^{m} v_{i}x_{ij}\leq0,j=1,2,\cdots,n,$$

$$\text{ }\sum_{r=1}^{s} v_{i}x_{ij}=1\quad\quad u_{r}\geq0,v_{i}\geq0, \forall r,i.$$

Output oriented CCR model：

$$Minimize \sum_{i=1}^{m} v_{i}x_{io}$$

$$subject to \sum_{r=1}^{s} u_{r}y_{rj}-\sum_{i=1}^{m} v_{i}x_{ij}\leq0,j=1,2,\cdots,n,$$

$$\text{ }\sum_{r=1}^{s} u_{r}y_{ro}=1\quad\quad u_{r}\geq0,v_{i}\geq0, \forall r,i.$$

BCC model relaxes the assumption that the scale return of CCR model is constant.

Input oriented CCR model：

$$Maximize\text{ }\sum_{r=1}^{s} u_{r}y_{ro}-u_{o},$$

$$subject to\text{ }\sum_{i=1}^{m} v_{i}x_{io}=1,$$

$$\text{ }\sum_{r=1}^{s} u_{r}y_{rj}-\sum_{i=1}^{m} v_{i}x_{ij}-u_{o}\leq0,\text{ }j=1,\ldots,n,$$

$\text{ }u_{r},v_{i}\geq0,r=1,\ldots,s; i=1,\ldots,m.$

Output oriented CCR model：

$$Minimize\text{ }\sum_{i=1}^{m} v_{i}x_{ij}+v_{o},$$

$$subject to\text{ }\sum_{i=1}^{m} u_{r}y_{ro}=1,$$

$$\text{ }\sum_{r=1}^{s} u_{r}y_{rj}-\sum_{i=1}^{m} v_{i}x_{ij}-v_{o}\leq0,\text{ }j=1,\ldots,n,$$

$$\text{ }u_{r},v_{i}\geq0,r=1,\ldots,s; i=1,\ldots,m.$$

2、The difference between input-oriented and output-oriented applications

The input-oriented model measures the ineffectiveness of the evaluated DMU from the perspective of input. It focuses on the degree to which the technical effective inputs should be reduced without reducing output. The output-oriented model measures the ineffectiveness of the evaluated DMU from the perspective of output. It focuses on the degree to which the technical effective outputs should be reduced without increasing input The extent of the increase.

The difference between input orientation and output orientation is explained by the simplified production environment of single input (x) and single output (y).

Figure 1 Schematic diagram of input orientation

o

y

B

C_1_

C_2_

C

A

M

x

D

From Figure 1, schematic diagram of input guidance principle, it can be seen that:

In the constant return on scale model(CRS), OB ray is the production frontier, B is the only effective DMU, C point is the invalid rate DMU, and the input invalid rate is reflected in CC_1_.

In the variable return on scale model (VRS), the curve MABD constitutes the production frontier, point C is the invalid rate DMU, and the input invalid rate is embodied in CC_2_.

Figure 2 Schematic diagram of output orientation

o

y

B

C_3_

C_4_

C

A

x

D

From the schematic diagram of output orientation in Figure 2, it can be seen that:

In the CRS model, OB ray is still the leading edge of production, B is the only effective DMU, point C is the invalid rate DMU, and the input invalid rate is reflected in CC_3_.

In the VRS model, ABD and D are parallel to the extension line of the X axis to form the production front, point C is the invalid rate DMU, and the input invalid rate is CC_4_.

o

y

B

C_1_

C_2_

C

A

M

x

D

C_3_

C_4_

Q

N

Figure 3 Schematic diagram of input-oriented and output-oriented efficiency

In Figure 3, the efficiency values of input-oriented and output-oriented are put together. We can see the relationship between them intuitively:

In CRS model, the efficiency value of input-oriented and output-oriented is equal. The input-oriented efficiency value of point C is QC_1_ / QC, and the output-oriented efficiency value is NC / NC_3_. It is easy to prove that two values are equal.

In VRS model, the efficiency value of input-oriented and output-oriented is not equal. The input-oriented efficiency value of point C is QC_2_ / QC, and the output-oriented efficiency value is NC / NC_4_.

The technical efficiency (TE) of CRS model includes the scale efficiency.

The pure technical efficiency (PTE) of VRS model does not include scale efficiency.

Scale Efficiency（SE），SE=TE/PTE。

The input oriented scale inefficiency rate is reflected in C_1_C_2_。

The output oriented scale inefficiency rate is reflected in C_3_C_4_。
